# The Assessment of the Effect of Nano Propolis Against Melanoma Cell Line, and Its Radio Sensitization Effect

**DOI:** 10.1002/fsn3.71577

**Published:** 2026-05-20

**Authors:** Nasim Valivand, Nematollah Gheibi, Azam Janati Esfahani, Hossein Ahmadpour_Yazdi, Hajie Lotfi, Shima Bidabad, Kimia Taebi, Maryam Karimi

**Affiliations:** ^1^ Student Research Committee Qazvin University of Medical Sciences Qazvin Iran; ^2^ Cellular and Molecular Research Center, Research Institute for Prevention of Non‐Communicable Disease Qazvin University of Medical Sciences Qazvin Iran; ^3^ Department of Medical Biotechnology, School of Advanced Technologies in Medicine Qazvin University of Medical Sciences Qazvin Iran; ^4^ USERN Office Qazvin University of Medical Sciences Qazvin Iran; ^5^ Clinical Research Development Unit, Velayat Hospital Qazvin University of Medical Sciences Qazvin Iran

**Keywords:** cell cycle assay, comet assay, melanoma, nano propolis, radio protection, scratch assay

## Abstract

This study aimed to investigate the effect of Nano Propolis (NP) on cell viability, pro‐apoptotic gene expression, anti‐metastatic activity, and radiosensitization in the A‐375 skin cancer cell line. The IC_50_ values of 85 and 60 μg/mL were determined for propolis extract (PE) and NP, respectively, in cellular and molecular experiments using A‐375 melanoma cells and Vero normal cells. To assess apoptosis, the expression levels of *Bax* and *Bcl‐2* genes were measured using quantitative reverse transcription polymerase chain reaction (qRT‐PCR). A scratch assay was used to assess the ability of NP and PE to reduce tumor cell migration. A cell cycle assay evaluated the effect of both formulations on cell proliferation after 48 h of incubation. An alkaline comet assay was used to measure DNA strand breaks after treatment with NP or Propolis Extract, combined with ionizing radiation. All statistical analyses were performed using SPSS v.20 and GraphPad Prism v.9. NP at its IC_50_ concentration showed a stronger ability to increase the apoptotic cell rate, inhibit cancer cell migration, suppress cancer cell growth through G1‐phase arrest, and increase radioprotective effects in the normal cell line, while also increasing radiosensitivity in the cancer cell line compared with PE. NP demonstrated better and more effective activity against A‐375 melanoma cells than PE.

## Graphical abstract

Iranian nano propolis stoppeded the cell cycle at G1 phase, increased the apoptosis and anti‐metastasis effect rather than propolis extract. The combination of Iranian nano propolis with ionizing radiataion significantly increased its anticancer properties on cancer cells and protected normal cells. The better and more effective performance of propolis nanoparticle was evident against melanoma cancer cells, A‐375, than propolis extract.

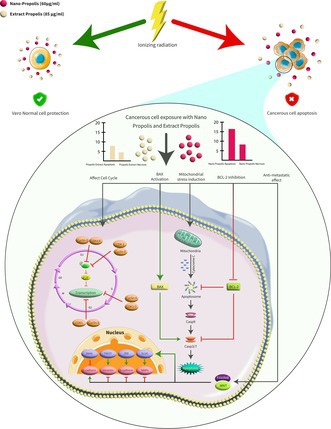

## 1 Introduction

Melanoma, the most lethal form of skin cancer, occurs in approximately 4% of individuals (Owida 2022) and, in addition to the skin, may also involve other mucosal surfaces, the eyes, and the meninges (Ali et al. 2013).

Its strong tendency to metastasize, even when the primary tumor is small, leads to an unfavorable prognosis. According to the GLOBOCAN 2020 report, there were 324,635 cases of melanoma worldwide, representing 1.7% of all cancers, and melanoma accounted for 0.6% of all cancer‐related deaths (Switzer et al. 2022).

Identifying the mechanisms underlying melanoma pathogenesis may lead to more substantial progress in therapeutic approaches. Over recent decades, several signaling pathways associated with melanoma have been described, including the mitogen‐activated protein kinase (*MAPK*) pathway, the protein kinase B (*AKT*) pathway, the cell cycle regulatory pathway, the pigment‐related pathway, the p53 pathway, epigenetic factors, and other related pathways (Guo et al. 2021). The survival rate of patients with skin cancer relates strongly to earlier diagnosis and effective treatment (Kurangi and Jalalpure 2018; Owida 2022).

Common treatment approaches for melanoma include surgery, immunotherapy, chemotherapy, and radiotherapy (Aghamohammadi et al. 2022). Unfortunately, surgery that aims to remove the tumor often does not improve patient survival. In addition, combination regimens that include chemotherapy cause many side effects in patients and contribute to multidrug resistance.

Herbal medicines such as Propolis may offer advantages over conventional chemical drugs because they can increase the effectiveness of standard chemotherapy and reduce its side effects when used as therapeutic supplements (Kurangi and Jalalpure 2018).

Propolis, collected by honeybees through mixing salivary enzymes with plant materials, is a complex substance that contains a wide range of chemicals and can exert various structural and biological effects on cancer cells. The chemical composition of Propolis varies with the hive's geographical location, local vegetation, and the season in which it is harvested (Kekeçoğlu et al. 2021). More than 500 compounds have been identified in Propolis (Luque‐Bracho et al. 2023). Many preclinical and clinical studies have examined natural constituents of Propolis, including flavonoids, phenolic compounds, polyphenols, terpenes, terpenoids, and aromatic acids, for their potential anticancer, antibacterial, antifungal, anti‐parasitic, antioxidant, and anti‐inflammatory activities, as well as their ability to support immune function (Aghamohammadi et al. 2022; Aravand et al. 2025; Gheibi et al. 2016; Huang et al. 2014).

Flavonoids and polyphenols inhibit cancer progression by blocking the MAPK and *Wnt*/β‐catenin pathways and by activating the *MEK–ERK signaling* pathway. These actions halt the cell cycle, trigger apoptosis and autophagy, and reduce cancer cell proliferation and invasion (Li et al. 2022; Valivand et al. 2024).

Nanotechnology, the science and technology of materials and systems at the nanoscale (1–100 nm), supports advances in human and animal health, environmental protection, food security, and disease control (Tatli Seven et al. 2018).

The use of Propolis in extract or free form limits its therapeutic activity because of low solubility, poor absorption, untargeted release, and low bioavailability (Houshang et al. 2012). In contrast, Nano Propolis (NP) shows markedly higher absorption due to its small size, greater reactivity, and higher solubility (Kumari et al. 2023).

Radiation therapy, a central modality in cancer treatment, uses ionizing radiation to destroy cancer cells by inducing DNA damage and disrupting cellular proliferation (Hall and Giaccia 2006). Studies report that Propolis extract increases the cytotoxic effects of radiotherapy on cancer cells through mechanisms such as impairing DNA repair, modulating apoptotic pathways, and reducing radiation‐induced oxidative stress (Yi et al. 2021).

This study aimed to investigate the effect of Iranian NP on proliferation, invasion, and the expression of *BAX, BCL2*, and *Caspase3*, as well as its radioprotective and radiosensitizing effects on the A‐375 cancer cell line and the normal Vero cell line.

The combination of radiotherapy and chemotherapy aims to improve therapeutic outcomes by maximizing tumor control while minimizing toxicity to healthy tissues. However, the effectiveness of these combined treatments often remains limited by drug toxicity, low solubility, and suboptimal timing. Nanotechnology provides potential solutions through the development of different nanocarriers, such as liposomes, polymeric nanoparticles, and inorganic nanoparticles, which increase drug solubility, reduce side effects, and improve tumor targeting via mechanisms such as the enhanced permeation and retention (EPR) effect. Functionalization of these nanocarriers with targeting molecules further increases their specificity and uptake by cancer cells. By allowing more efficient drug delivery and controlled release, nanotechnology may improve the efficacy of concurrent radiotherapy and chemotherapy (Denkova et al. 2020).

## 2 Material and Methods

### 2.1 NP Preparation

Propolis was mixed with 70% ethanol. The mixture was kept in a dark container with moderate shaking for 14 days. The extract was then filtered, freeze‐dried, and concentrated by rotary evaporation (Buchi, Switzerland). The chemical composition of Propolis was analyzed by X‐ray Diffraction (XRD) (Bruker, Germany). To prepare 8 mL of NP, 0.24 g of freeze‐dried ethanolic extract of Propolis was dissolved in 2 mL of 80% ethanol. For nanoparticle preparation, 0.12 g of freeze‐dried Propolis was dissolved in 1 mL of 80% ethanol in a microtube. The mixture was sonicated using an Elmasonic sonicator (Elmasonic, Germany) at 30 W and 30°C, with an on/off cycle of 3 s/8 s for 35 cycles. The final nanoparticle dispersion contained 960 μL of Propolis, 960 μL of a Tween 80/20 mixture, and 2080 μL of distilled water. To prevent evaporation, sonication was performed on ice.

After 35 cycles, the mixture was further sonicated with an on/off cycle of 4 s/6 s for 5 min, followed by 7 min of rest. The formulation was considered stable when mixing 500 μL of the nanoparticle solution with 500 μL of distilled water did not produce turbidity. No sedimentation was observed after centrifugation at 14,000 rpm for 10 min, and no aggregation was visible under a light microscope. NP was characterized by measurement of dynamic light scattering (DLS) and zeta potential.

### 2.2 Cell Culture

The melanoma cell line A‐375 and the normal kidney cell line Vero were obtained from the Iranian Biological Resource Center (Tehran, Iran). Cells were cultured in DMEM supplemented with 10% FBS and 1% penicillin/streptomycin (1000 U/mL penicillin, 1000 μg/mL streptomycin) at 37°C in a humidified incubator with 5% CO₂. Cultures were maintained as adherent monolayers.

### 2.3 Cell Irridation

A‐375 and Vero cells in Petri dishes were exposed to a 4 Gy dose of 6 MV X‐rays and 6 MeV electrons using an Elekta Versa HD linear accelerator (Velayat Hospital, Qazvin, Iran). Irradiation was performed at a source‐to‐surface distance (SSD) of 100 cm with a field size of 20 × 20 cm^2^. A 1‐cm‐thick water bolus was placed on top of the cell plates as a build‐up material. Non‐irradiated samples served as controls.

### 2.4 Cellular Assay

#### 2.4.1 Cell Viability Assay

This experiment assessed the cytotoxicity of PE and NP using the MTT assay, following the method described previously by Sabaghi et al. (Sabaghi et al. 2024). First, 5000 A375 cells in 200 μL of cell suspension were seeded into each well of a 96‐well plate and cultured for 24 h. Cells were then exposed to varying concentrations (10, 20, 40, 60, 70, 80, 90, and 100 μg/mL) of the extract or NP for 24 and 48 h.

For the preparation of NP, 0.12 g of Propolis was added to 4 mL, corresponding to 0.03 g of Propolis in 1 mL and a stock concentration of 30,000 μg/mL. For preparation of the working concentrations, equation 2 was used, where 30,000 μg/mL represents M1, the unknown quantity represents V1, the desired concentration represents M2, and the volume required for each well represents V2. For example, for a concentration of 100 μg/mL, the calculation proceeds according to this equation:

M1V1=M2V2

Equation 2. Preparation of different concentrations.

30,000* V 1 = 100*200.

V 1 = 0.6λ.

Control groups consisted of culture medium and 15% DMSO for negative and positive controls, respectively. For the cytotoxicity assays, 15% DMSO served as the positive control. Although standard positive controls usually include 1%–2% Triton X‐100 or approximately 10% DMSO, in this experimental setting, these concentrations did not induce complete cytotoxicity. Therefore, 15% DMSO was selected to ensure a consistent and strong cytotoxic effect.

The experiment was conducted three times, and the IC_50_ values were determined using GraphPad Prism v.9 (GraphPad Software Inc., United States).

#### 2.4.2 Flow Cytometry Test

Apoptosis was assessed by treating A‐375 cells with the specified concentrations of extract and Propolis NPs, followed by flow cytometry using a staining kit containing Annexin V‐FITC and PI (Bioscience Cell Apoptosis Kit, IQ Products, Annexin V Apoptosis Detection Kit FITC). Following the manufacturer's protocol, A‐375 cells were seeded in 6‐well plates and treated with PE and NP for 48 h at 37°C. After harvesting, cells were washed with PBS and stained with Annexin V‐FITC for 30 min and PI for 5 min. Data were analyzed using FlowJo v10.5.3 software.

#### 2.4.3 In Vitro Scratch/Wound Healing Assay

Migration assays are suitable for assessing the ability of a drug or compound to reduce tumor cell metastasis.

A‐375 cells were placed in 12‐well plates with a density of 3 × 10^5^. After reaching 80% confluency, cells were treated with the IC_50_ concentration of PE and NP. The control group received no treatment.

A vertical scratch was then made in each well using a plastic pipette tip. Cell movement into the scratched area was recorded with an OPTIKA phase‐contrast microscope (B‐380 Series, Italy) at 0, 4, 8, 12, 24, and 48 h, and the resulting images were analyzed using AutoCAD and Excel software.

#### 2.4.4 Cell Cycle Assay

To assess the effect of extract and Propolis NPs on the cell cycle, 3 × 10^5^ A‐375 cells were seeded into 6‐well plates and incubated for 24 h at 37°C. Cells were then exposed to the IC_50_ concentrations of extract and nanoparticles for 48 h at 37°C. After centrifugation (1500 rpm for 5 min) and two washes with PBS, 250 μL of a master mix solution (containing 20 μg/mL RNase A and 50 μg/mL PI) was added, and samples were incubated at 37°C for 1–2 h. DNA content was measured using a BD FACSCalibur (BD Biosciences, San Jose, CA, USA) by detecting PI fluorescence in the FL3 (red) channel, and data were analyzed using FlowJo v10.5.3 software.

#### 2.4.5 Alkaline Comet Assay

To assess DNA strand breaks, 3 × 10^5^ A‐375 and Vero cells were seeded in Petri dishes and treated with the IC_50_ concentrations of extract and Propolis NPs. Cells were then exposed to 6 MeV electron radiation (4 Gy) and 6 MV photon radiation (4 Gy) using a linac (Elekta, Versa HD).

Single‐ and double‐strand DNA breaks were evaluated using the alkaline comet assay, according to the method described previously by Fazeli et al. (Fazeli et al. 2007). Approximately 100 cells per slide were examined, and the mean tail moment was calculated as an indicator of DNA damage.

### 2.5 Molecular Assay

#### 2.5.1 Evaluation of Apoptotic Gene Expression

Total RNA was extracted from control and treated A‐375 cells using the RNA Fast Total RNA Extraction Kit (RNA Fanavaran, Tehran, Iran), according to the method described previously by Bahrami et al. (Bahrami et al. 2024). The qRT‐PCR conditions and gene‐specific primers are shown in Tables 1 and 2, with GAPDH used as the internal control.

**TABLE 1:** Sequence of primers of genes *BCL‐2*, *BAX*, and GAPDH.

Gene	Reverse	Forward
*BAX*	5′‐CTCAGCCCATCTTCTTCC‐3′	5′‐GCCTCCTCTCCTACTTTG‐3′
*BCL‐2*	5′‐GCATTCTTGGACGAGGG‐3′	5′‐TGGGAAGTTTCAAATCAGC‐3′
*Caspase‐3*	5′‐ACCGAGCTCCGAGGGCGGGAG‐3′	5′‐GCGGTAGCGCCGTCCGTTGC‐3′
*GAPDH*	5′‐TGGAAGATGGTGATGGGATT‐3′	5′‐CAATGACCCCTTCATTGACC‐3′

**TABLE 2:** Temperature–time program of *BAX*, *BCL‐2*, and *caspase‐3*.

Stage	Cycle	Temperature (°C)	Time	Genes (BAX, BCL2)	Genes (CASPase3)
Initial denaturation	1 cycle	95	5 min	✔	—
			7 min	—	✔
Denaturation	40 cycles		40 s	✔	—
			30 s	—	✔
Annealing		57	40 s	✔	—
		56	30 s	—	✔
Extension		72	40 s	✔	—
			20 s	—	✔

To assess expression changes of *BAX, Bcl‐2*, and *Caspase‐3* in cells treated with PE and nanoparticles, RNA was extracted from A‐375 cells. A NanoDrop spectrophotometer was used to measure RNA concentration and purity, and to confirm the absence of DNA, protein, and other solutes in the samples. A NanoDrop device was also used to evaluate the concentration and purity of the synthesized cDNA. To determine the optimal primer annealing temperature, the thermocycler was programmed with a temperature gradient using one cDNA sample for each of the *BAX*, *BCL‐2*, *Caspase‐3*, and *GAPDH* genes, followed by electrophoresis.

A real‐time PCR system recorded melting curves and Ct values for each sample. The *Pfaffel* method was used to calculate the fold change for each gene.

### 2.6 Statistical Analysis

Each experiment was performed in triplicate. A one‐way ANOVA was used to evaluate differences between the treated and control groups. Duncan's multiple‐range test, *t*‐test, F‐test, and Z‐scores indicated significant differences between data groups. Analyses were conducted using SPSS version 20 (SPSS, Armonk, NY, USA), with a significance threshold of *p* < 0.05. Graphs were generated using GraphPad Prism 9.

## 3 Results

### 3.1 Analysis of Nanoparticles

**FIGURE 1:**
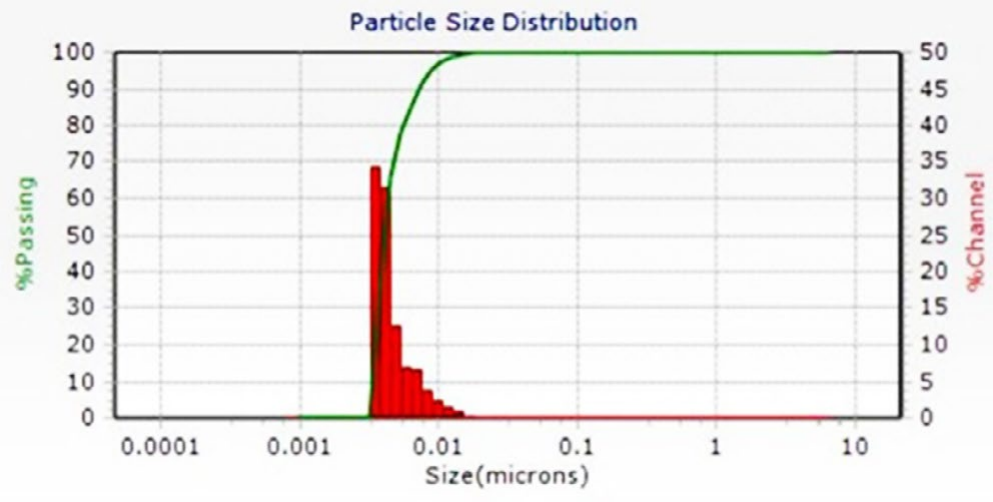
DLS size analysis of PNPs by sonicator method.

The DLS analysis of NP size distribution showed that the nanoparticles measured 4.1 ± 2.7 nm (Figure 1). Zeta potential measurements indicated a surface charge of −33 mV.

### 3.2 Cellular Test Results

#### 3.2.1 Cell Viability Assay

To assess the cytotoxic effects of PE and NP on the A‐375 cell line, cells were exposed to concentrations of 10–100 μg/mL for 24 and 48 h. As shown in Figure 1, treatment produced a significant, concentration‐dependent decrease in A‐375 cell viability. The greatest and smallest reductions in cell viability relative to the control occurred at 100 and 10 μg/mL, respectively, for both treatments after 48 h.

**FIGURE 2:**
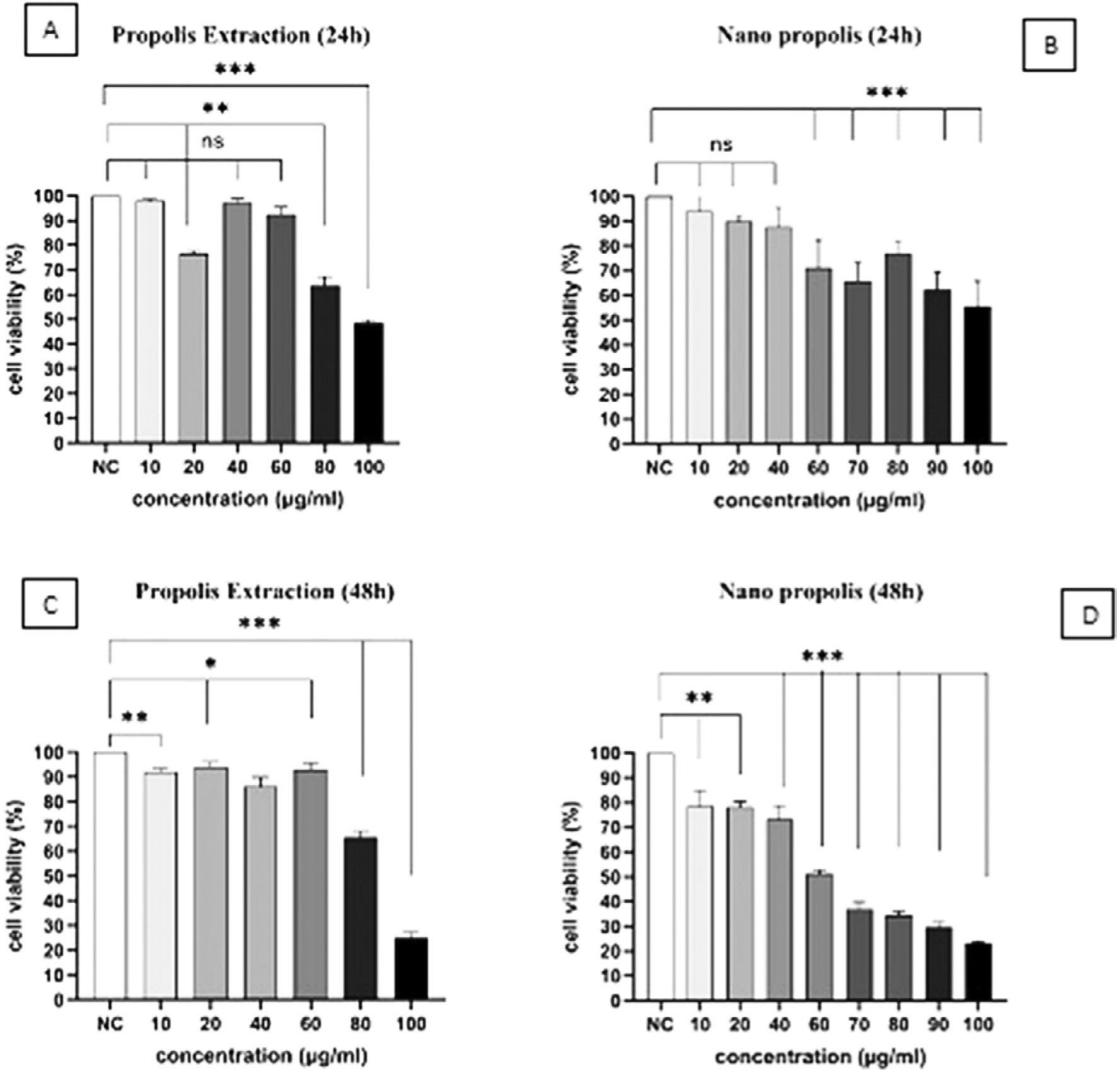
Analysis of MTT data obtained from A‐375 cells treated with PE and NP, for 48 h. (A) Cells treated by PE in 24 h; (B) Cells treated by PNPs in 24 h; (C) Cells treated by PE in 48 h; (D) Cells treated by PNPs in 48 h. (*p*‐values under 0.0001, 0.001, and 0.05 were considered significant. NC, negative control) *: *p* < 0.05, **: *p* < 0.01 and ***: *p* < 0.001.

**FIGURE 3:**
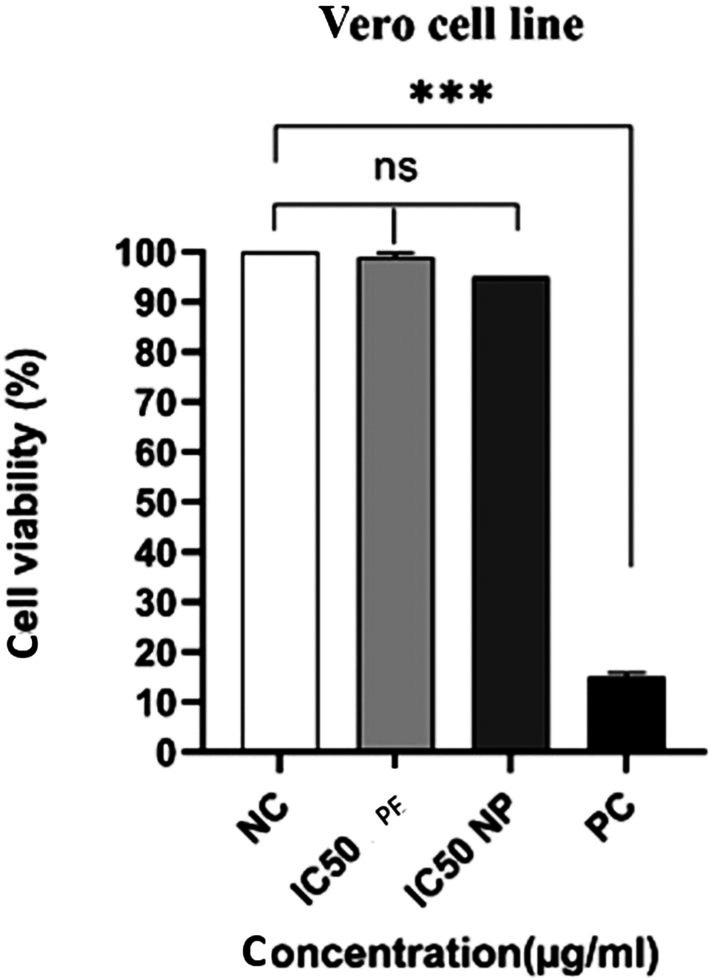
Analysis of MTT assay of normal Vero cells treated with IC_50_ concentration of PE and NP, in 48 h. (***: *p*‐value under 0.0001 was considered significant.) (NC, negative control; PE, propolis extraction; NP, nano propolis; PC, positive control).

The IC_50_ values for PE and NP were 84.92 and 103.14 μg/mL, respectively, for 24 h (Figure 2A,B), and the closest doses to the IC_50_ were calculated as 85.33 and 59.26 μg/mL for PE and NP, respectively, for 48 h (Figure 2C,D). The cytotoxic effects suggest that PE and NP may induce apoptosis in A‐375 cells. At the 48 h IC_50_ concentrations for A‐375 cells, normal Vero cells showed no significant cytotoxic response (Figure 3).

Therefore, the cellular and molecular mechanisms were investigated using flow cytometry, wound‐healing assays, cell‐cycle analysis, gene‐expression profiling, the alkaline comet assay, and protein–protein interaction analysis of *BAX*, *Bcl‐2*, and *Caspase‐3*. These results were compared with the cytotoxic effects of the two compounds. The effects of the PE and NP concentrations on A‐375 cells were assessed after 48 h of treatment.

#### 3.2.2 Flow Cytometry Test Analysis

**FIGURE 4:**
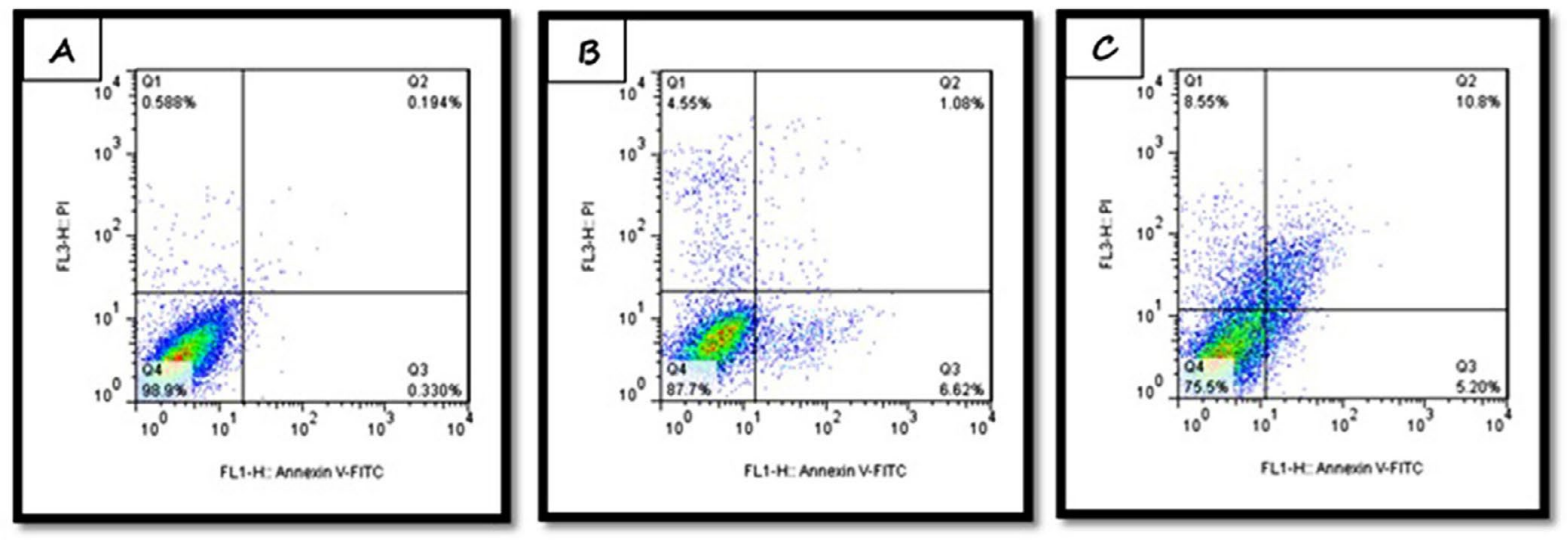
The investigation of necrotic and apoptotic A‐375 cancer cells treated by PE and NP in 48 h. (A) Control cells (without treatment); (B) Cells treated by PE in 48 h; (C) Cells treated by NP in 48 h.

**FIGURE 5:**
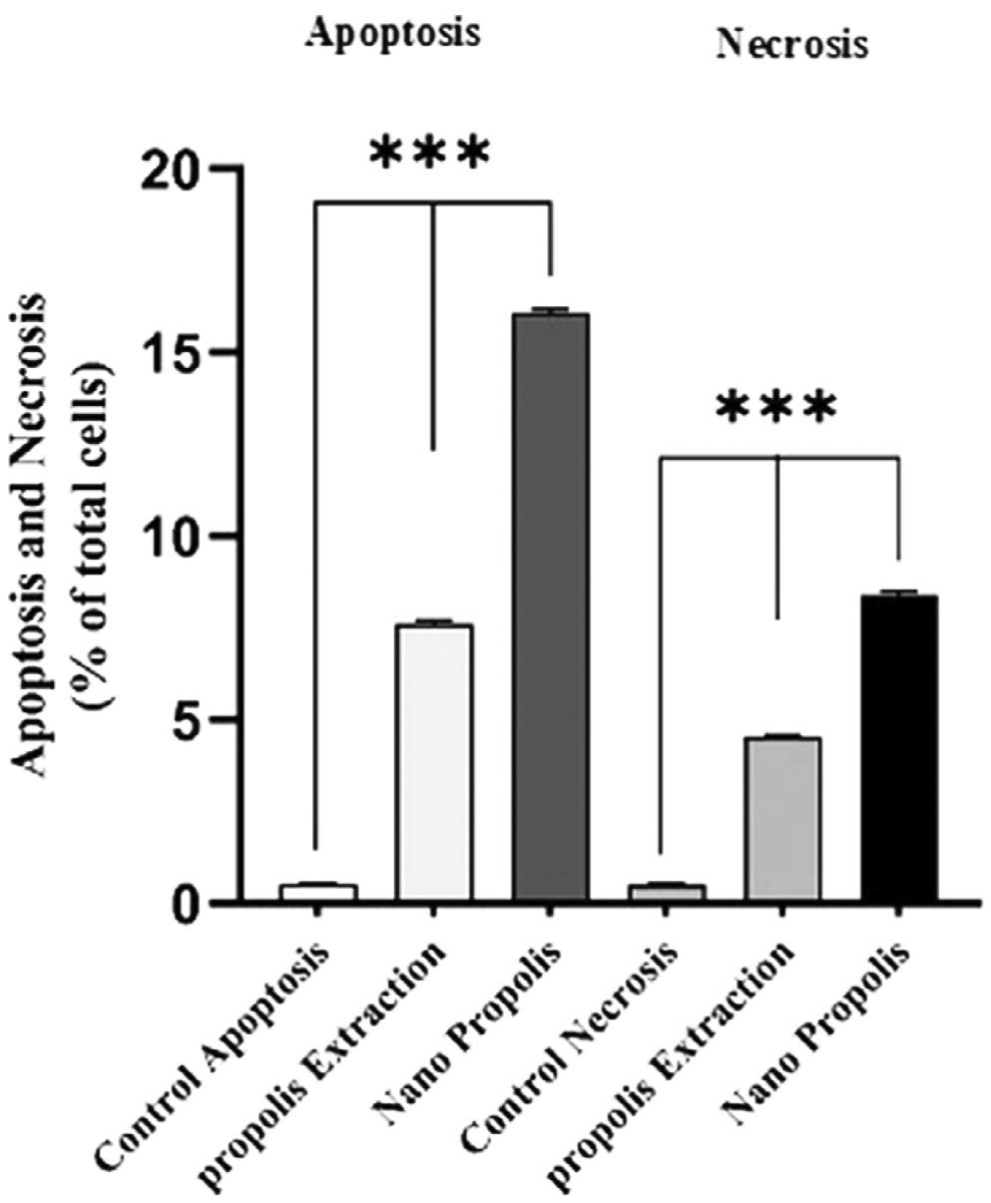
Quantitative graph of apoptosis and necrosis percentage in A‐375 cells treated by IC_50_ concentration of PE and NP in flow cytometry test. The highest apoptosis and total necrosis percentage were observed in cells treated with PNPs. (***: *p*‐value under 0.0001 was considered significant.)

Flow cytometry measured the percentages of viable, necrotic, and apoptotic cells after treatment of A‐375 cells with PE and NP for 48 h (Figure 4). Both treatments increased the proportion of apoptotic cells compared with the control group. The total apoptotic cell rate was 7.7% for PE and 16% for NP, with NP inducing the highest level of apoptosis relative to both the control and the extract (Figure 5).

#### 3.2.3 Wound Healing (Scratch) Assay

**FIGURE 6:**
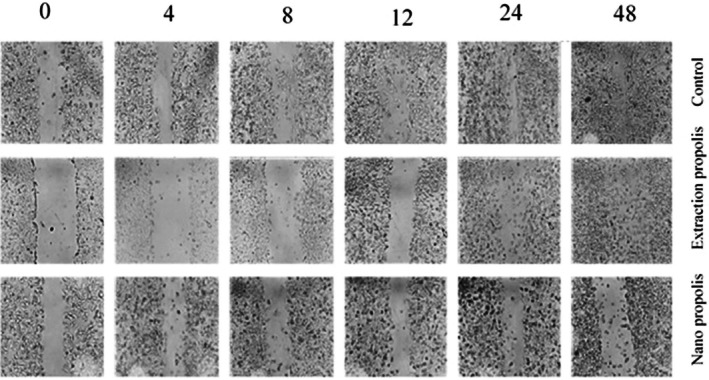
Pictures of A‐375 cancer cells treated with PE and NP in a 0, 4, 8, 12, 24, and 48‐h period for wound healing (scratch) assay; the first row corresponds to untreated cells (control); the second row for cells treated with PE and the third row for cells treated with NP.

An inverted microscope was used to analyze scratch width at different time points and to assess the effects of PE and NP on A‐375 cell migration. In the control group, the scratch progressively narrowed and eventually closed over 0, 4, 8, 12, 24, and 48 h. The surface area calculated from the images confirmed this reduction. The Pearson correlation coefficients for the control and PE groups were −0.84 and −0.89, respectively, indicating an inverse relationship between time and scratch width. In contrast, in the NP‐treated group, the scratch width did not decrease, and the correlation coefficient was positive (Figure 6). These findings suggest that NP, at its IC50 concentration, is more effective than PE at inhibiting cancer cell migration.

#### 3.2.4 Cell Cycle Assay Results

**FIGURE 7:**
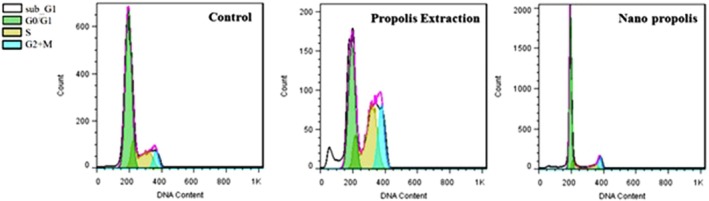
DNA content analysis in A‐375 cells after treatment with IC_50_ concentrations of PE and NP during 48 h using flow cytometry.

The cell cycle comprises sequential processes regulated by checkpoint proteins. Normal differentiated cells typically remain in the G0 phase (Figure 7), where they stop dividing. Some cells re‐enter the cycle and progress through interphase and mitosis to produce two daughter cells. Others enter G0 and stay inactive until they receive an external signal that triggers entry into G1. In this study, cell cycle analysis was performed on cells treated with PE and NP at their IC_50_ values for 48 h.

**FIGURE 8:**
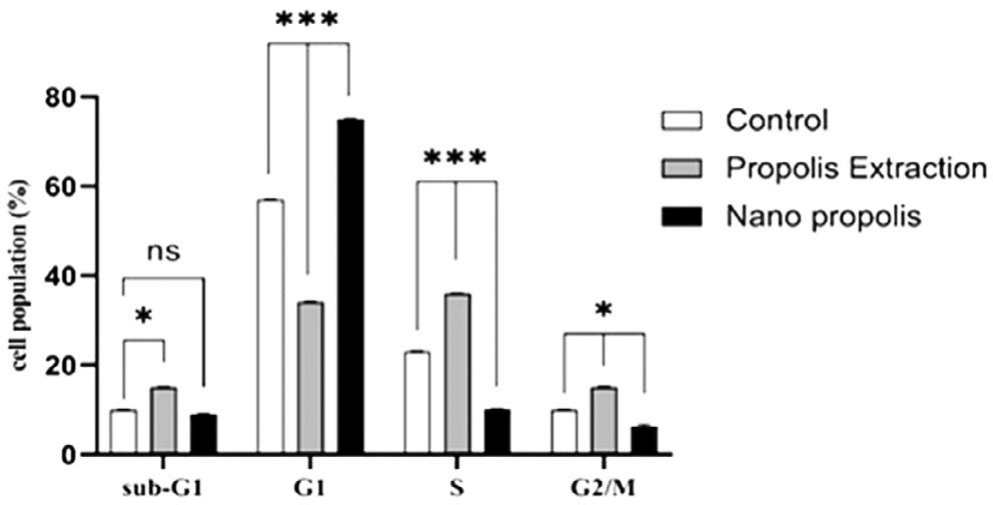
Quantitative analysis of cell cycle in A‐375 cells after treatment with PE and NP at IC_50_ concentration. (***: *p*‐values under 0.001, **: under 0.01, and *: under 0.05 were considered significant.)

The results shown in Figure 8 indicate that treatment with PE increased the cell populations in the S, M/G2, and sub‐G1 phases, whereas the G1‐phase population decreased. In contrast, cells treated with NP showed a reduction in S‐ and M/G2‐phase populations and an increase in the G1‐phase population compared with the other groups. These findings suggest that NP may inhibit cancer cell growth and proliferation by arresting the cell cycle in G1.

#### 3.2.5 Alkaline Comet Assay Analysis

**FIGURE 9:**
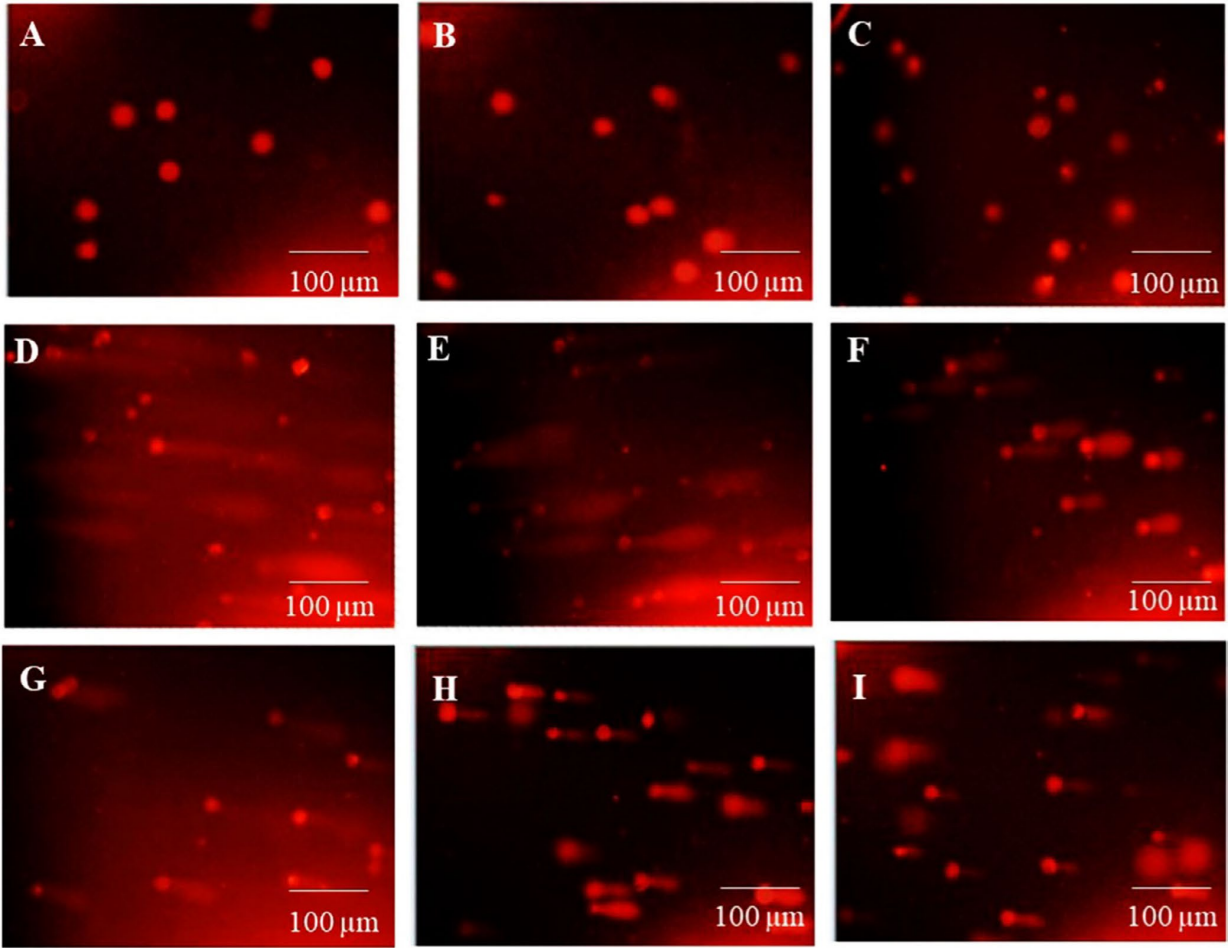
Microphotography of alkaline comet assay of normal Vero cells by fluorescence microscopy. The first row for cells without radiation, the second row with photon radiation, and the third row with electron radiation. (A, D, G) control cells; (B, E, H) cells treated with PE; (C, F, I) cells treated with NP.

**FIGURE 10:**
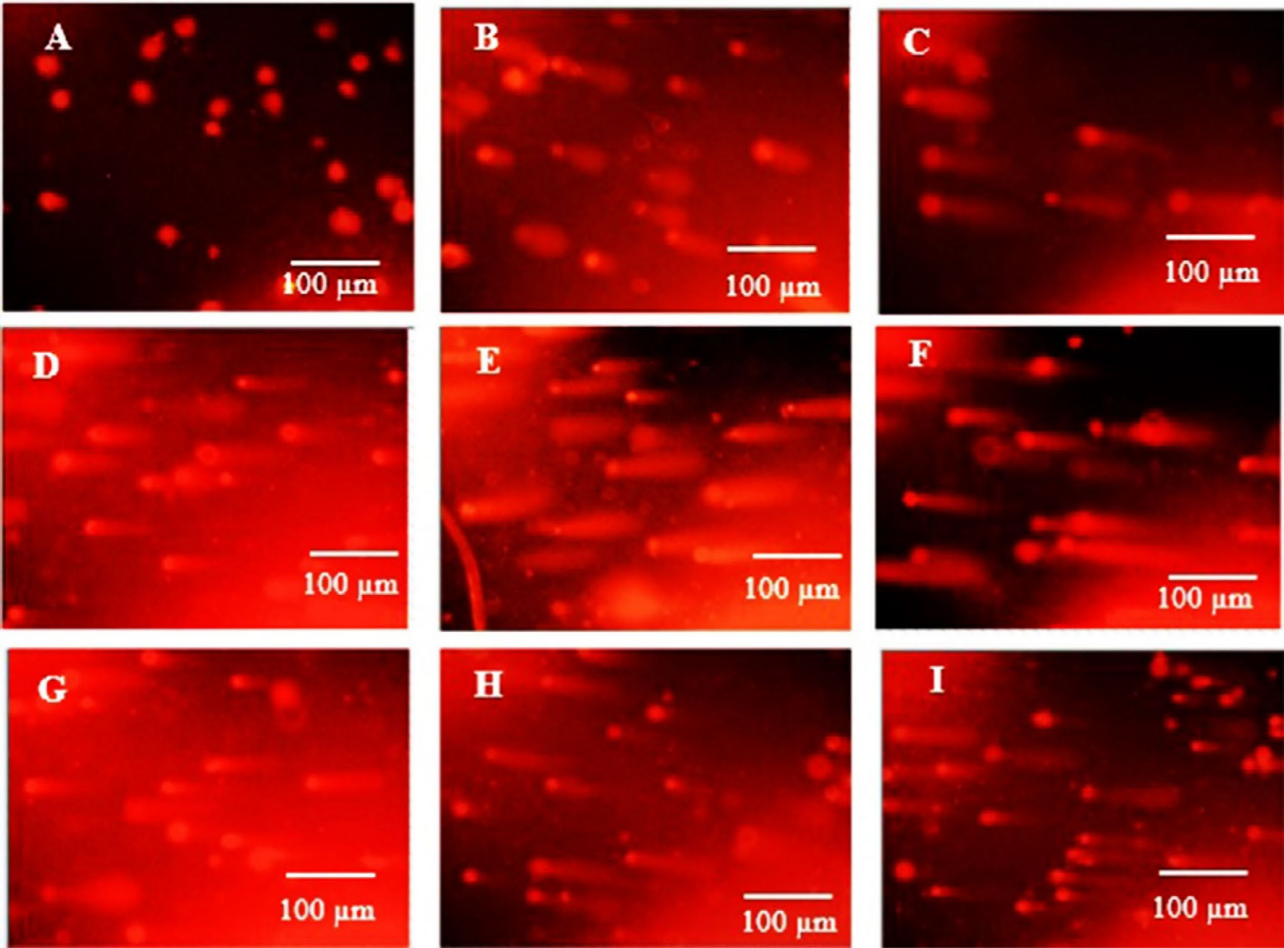
Microphotography of alkaline comet assay of A‐375 cells by fluorescence microscopy. The first row for cells without radiation, the second row with photon radiation, and the third row with electron radiation. (A, D, G) control cells; (B, E, H) cells treated with PE; (C, F, I) cells treated with NP.

The alkaline comet assay was performed on both the Vero cell line and the A‐375 cancer cell line after exposure to electron and photon radiation. Cells treated with the IC_50_ concentrations of PE and NP were evaluated 48 h after exposure. Micrographs of the alkaline comet assay for A‐375 and normal Vero cells were obtained by fluorescence microscopy (Figures 9 and 10). Tail moment values were used to quantify the extent of DNA damage.

**FIGURE 11:**
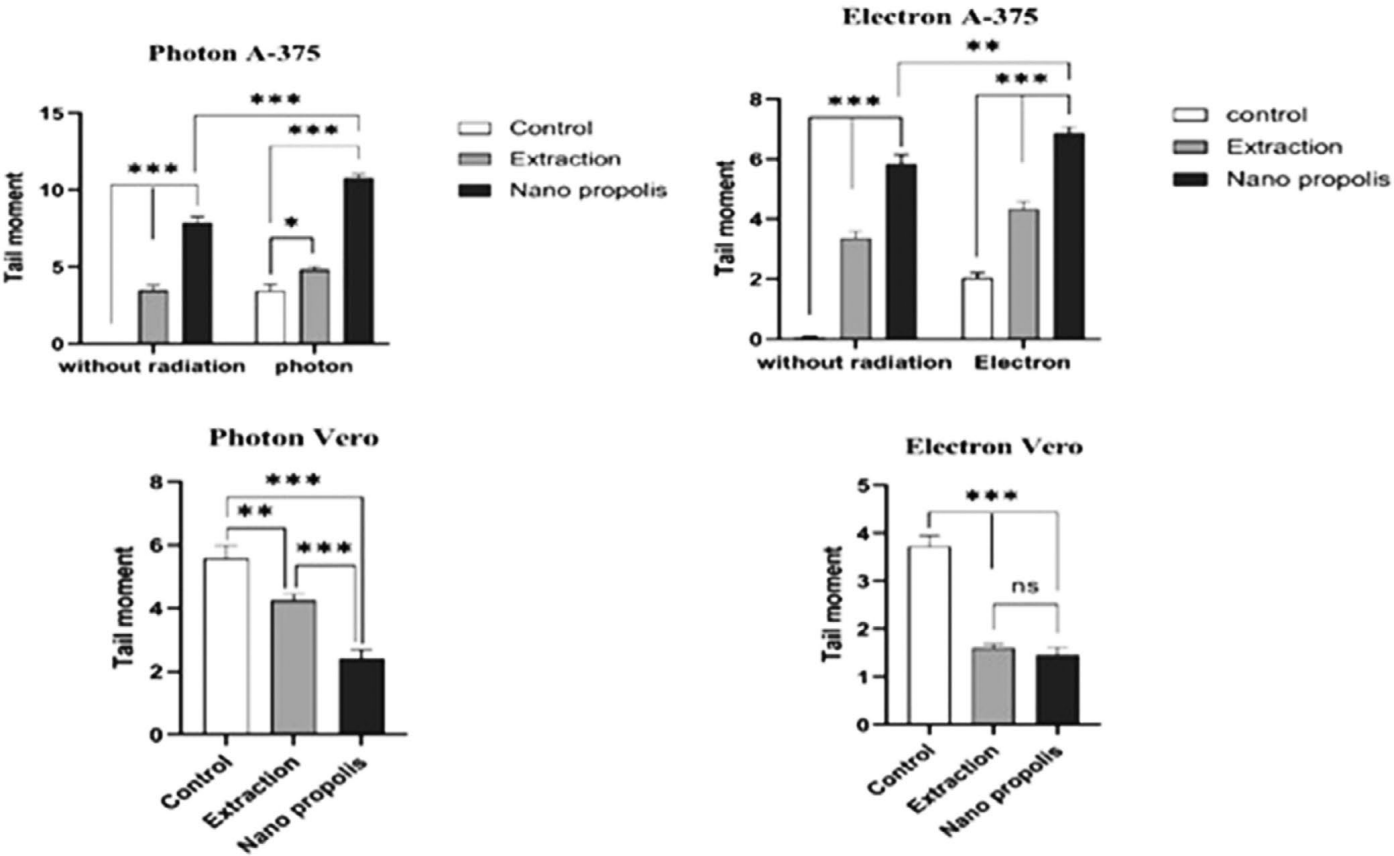
The effect of radiation treatment (RT) plus extraction/nano Propolis on DNA damages of A‐375 and Vero cells from monolayer culture. A‐375 cancer cells (A and B) and normal Vero (C and D). *: *p‐*value < 0.05, **: *p*‐value < 0.01 and ***: *p*‐value < 0.001.

The MTT results showed that Propolis treatments did not cause significant toxicity in Vero cells, and no comet tail was observed in the control samples (untreated, extract, and Propolis nanoparticles without radiation). In contrast, in normal Vero cells exposed to photon and electron radiation, IC_50_ values for PE and NP reduced the tail moment via a radioprotective effect relative to the irradiated control group. For photon exposure, tail moment values were 5.65, 4.42, and 2.62 for the control, PE, and NP groups, respectively (Figure 11). For electron exposure, the corresponding values were 3.60, 1.75, and 1.60. These findings indicate that Propolis exerted a radioprotective effect in the normal cell line, reduced DNA damage after irradiation, and helped prevent injury to healthy tissues.

In A‐375 cells, comets appeared in the non‐irradiated groups, and tail moment values increased further after photon and electron exposure. This pattern confirms the radiosensitizing effect of Propolis in the cancer cell line. For photon irradiation, tail moment values before and after exposure were 0 and 3.80 for the control, 3.80 and 4.80 for PE, and 1.80 and 11.00 for NP, respectively. For electron irradiation, the corresponding values were 0 and 2.16 for the control, 3.20 and 4.16 for PE, and 4.70 and 6.32 for NP.

### 3.3 Molecular Assay

**FIGURE 12:**
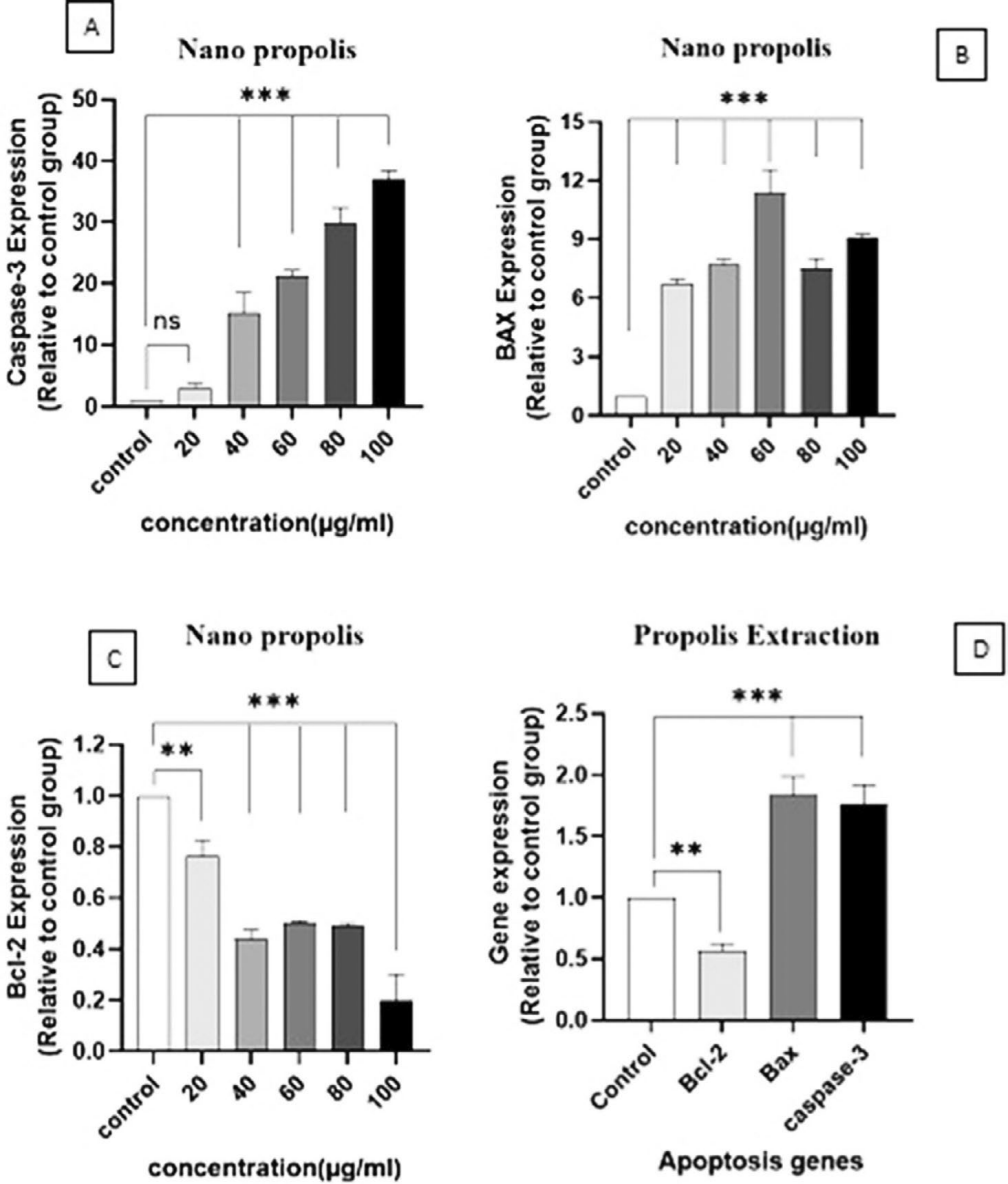
The expression analysis of Caspase‐3, *BAX*, and *BCL‐2* genes using *P‐ffafel's* formula. Quantitative analysis of qRT‐PCR results of A‐375 cells treated with NP at IC_50_ concentration and two lower and two higher concentrations on gene expression of (A) Caspase‐3, (B) *BAX*, (C) *Bcl‐2* in 48 h, (D) Quantitative analysis of qRT‐PCR results of A‐375 cells treated with IC_50_ concentration of PE of 48 h treatment time. *** :*p*‐values under 0.001 and **: under 0.01 were considered significant.

**FIGURE 13:**
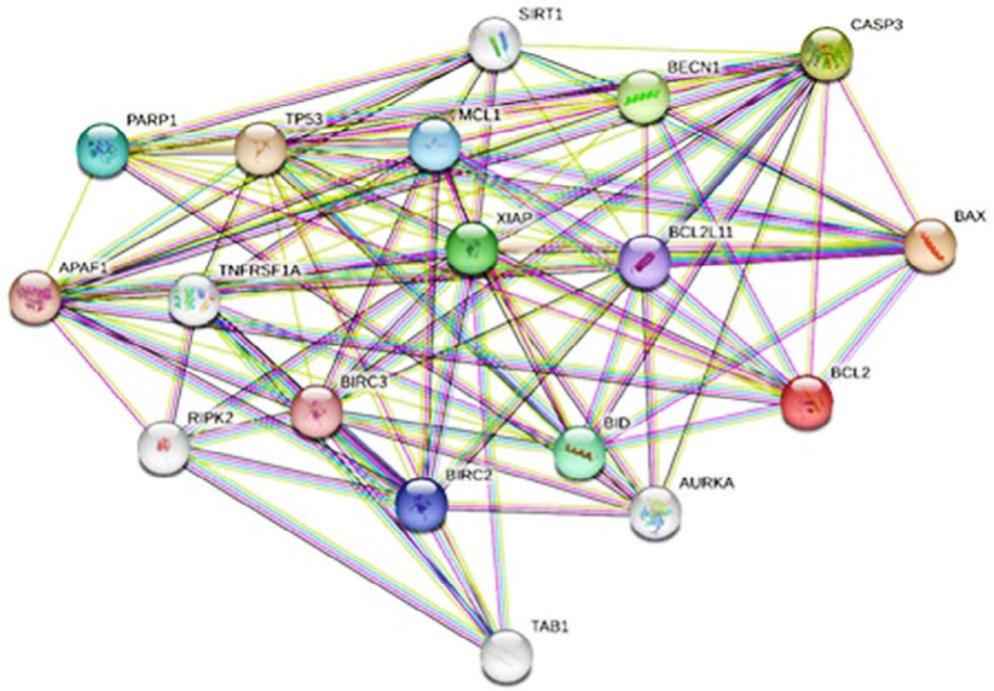
Network of *BAX*, BCL2, and *Caspase‐3* genes.

The fold change values, indicating gene expression changes compared with the control group, and their significance levels for *BAX*, Caspase‐3, and *BCL‐2* are presented in Figure 12. The results indicate that both NP and PE significantly enhance the expression of *Caspase‐3* and *BAX* genes (Figure 12A,B) while reducing the expression of the *Bcl‐2* gene (Figure 12C). NP also showed a greater effect on gene expression than PE (Figure 12D). Analysis of protein–protein interactions using the STRING database (Figure 13) indicates that *Caspase‐3*, *BAX*, and *Bcl‐2* are part of a common interaction network.

## 4 Discussion

This study showed that NP exhibits stronger anticancer effects than PE, as reflected by a lower IC_50_ after 48 h of treatment. The nanoparticles, with diameters below 10 nm and a negative surface charge, exhibited high stability. Both NP and PE induced apoptosis in melanoma cells through changes in apoptosis‐related genes. At the same time, NP increased the sensitivity of cancer cells to radiotherapy and conferred protective effects on normal cells. These findings suggest that Propolis, particularly in nanoparticle form, is a promising natural candidate for adjuvant cancer therapy, combining potent antitumor activity with selective cytoprotection.

Plant‐derived natural compounds, such as Propolis, have been studied as medicinal supplements for their potential anticancer effects and their ability to regulate multiple signaling pathways (Safarzadeh et al. 2014; Valivand et al. 2024).

Nanotechnology has emerged as a promising approach to increase the efficacy and safety of therapeutic and diagnostic agents (Zhang et al. 2008). Iranian Propolis from different regions contains high levels of flavonoid and phenolic compounds (Yaghoubi et al. 2007).

This study aimed to compare the effects of PE and NP on proliferation and expression of apoptosis‐related genes in A‐375 melanoma cells. The surface charge of nanoparticles influences their cellular uptake and accumulation; negatively charged nanoparticles tend to undergo less clearance by the reticuloendothelial system and therefore exhibit longer circulation times (Salatin et al. 2015). In this study, DLS and zeta potential analyses were used to assess the size and surface charge of the NPs. The results showed that the nanoparticles were less than 10 nm in diameter and carried a negative surface charge, indicating long‐term stability.

The MTT assay after 48 h of treatment showed that the ethanolic Propolis extract significantly reduced A‐375 cell survival at 85 μg/mL, which was determined to be the IC_50_ for the extract. The IC_50_ values of PE vary with propolis content across regions. After preparation and characterization of NP, the IC_50_ of NP after 48 h of treatment was approximately 60 μg/mL, which is lower than the IC_50_ for PE. This reduction in IC50 likely reflects the increased chemical activity and reactivity of compounds in nanoparticle form, consistent with other studies (Asha and Narain 2020; Xu et al. 2018). In this study, two concentrations, 85 and 60 μg/mL, were therefore selected as the IC_50_ values for PE and NP, respectively, for subsequent cellular and molecular experiments.

Flow cytometry was used to measure apoptosis and necrosis in cells treated with the IC_50_ concentrations of PE and NP. The sum of early and late apoptosis for PE was 6.87%, and necrosis was 5.54%. For NP, apoptosis increased to 16% and necrosis to 5.8%. Compared with the control group (apoptosis 0.5%, necrosis 0.5%), both treatments produced a marked increase in cell death. The higher percentage of apoptosis with NP than with PE indicates a stronger effect of the nanoparticles on A‐375 cancer cells. In Yufei Zheng's study, the percentage of apoptosis in A‐375 cells treated with Chinese Propolis at 112 μg/mL reached 27% (Zheng et al. 2018).

In another 2010 study, Indonesian Propolis induced 13% apoptosis in MCF‐7 cells (Sawah and Kav 2010). Soltaninejad reported apoptosis rates of approximately 20%–22% after 48 h for Kermanshahi Propolis at 200 μg/mL in three cell lines, MDA‐MB‐231, SKBR‐3, and MCF‐7 (Soltaninejad et al. 2020). In Metomura's 2008 study, U937 cells treated with PE at 100, 300, and 500 μg/mL showed apoptosis rates of 4%, 10%, and 14%, respectively (Motomura et al. 2008). The higher percentages of apoptosis reported for PE in these studies, compared with the present work, likely reflect differences in the Propolis source.

To confirm the anti‐metastatic effect of PE and NP, a wound healing assay was performed on A‐375 cell monolayers before and after treatment with PE and NP. Qualitative observation under an inverted microscope and quantitative analysis of the wound area showed that A‐375 cells treated with NP could not repair the scratch from 0 to 48 h. In contrast, cell migration into the wound area occurred in both the control and PE groups. The effect of PE on migration was not significant compared with the control group. In contrast to the present results, in the study by Xuan et al. (2014), Chinese Propolis reduced the metastasis of treated MDA‐MB‐231 cells by almost 40% after 48 h (Xuan et al. 2014). One reason for the variable effects of Propolis is its composition, which varies with geographical origin and harvest time; the specific components present in each Propolis sample may activate different mechanisms in different cancer cell lines.

Ionizing radiations cause cell death by damaging DNA (Mahdavi et al. 2017). In radiation therapy, preserving healthy tissues around the tumor remains a major challenge. Identifying a radiosensitizer for tumor cells that also protects healthy tissue is highly valuable. In this study, Iranian NP met this expectation. NP showed dual effects on normal and cancer cells by exhibiting both radioprotective and radiosensitizing properties. Propolis reduced radiation‐induced damage in healthy tissues. In contrast, in cancer cells, Propolis increased radiation sensitivity, thereby supporting radiotherapy. Other studies have also reported similar effects of Propolis on cells (Ibáñez et al. 2023). For example, the study by Roberto et al. (Roberto et al. 2016), which evaluated the genotoxic effects of Brazilian Propolis, showed that Propolis acted as a radioprotective agent in the rat hepatoma cell line HTC.

The findings of Benzoic et al. indicate that administering Propolis and related flavonoids to mice before gamma‐ray (Co‐60) irradiation protected them from lethal effects associated with DNA strand breaks caused by ionizing radiation (Benković et al. 2008). Wahyuni et al. reported antioxidant properties of Propolis in human liver cells, as confirmed by the Comet assay (Wahyuni et al. 2021).

This study examined three key genes, *BAX*, *Bcl‐2*, and *Caspase‐3*, to evaluate apoptosis. The results showed that Propolis increased BAX and Caspase‐3 expression in A‐375 cells, thereby promoting cytochrome c release into the cytosol and supporting apoptosis. In addition, the observed decrease in *BCL‐2* expression, an anti‐apoptotic protein, indicates that PE and NP can induce apoptosis in cancer cells. When comparing PE and NP, cells treated with nanoparticles showed higher expression of pro‐apoptotic genes, whereas the reduction in BCL‐2 levels was similar for both treatments compared with the control.

The decline in *Bcl‐2* expression was not observed with increasing concentrations of NP (Figure 12). In Oliveria's 2022 study, the results showed that treatment with Portuguese Propolis in A‐375 cells increased the expression of apoptosis‐related genes such as *BAX*, Caspase‐3, Caspase‐9, and *p53*, while reducing anti‐apoptotic proteins like *Bcl‐2* and *XL‐Bcl*, thereby promoting apoptosis in melanoma cells (Oliveira et al. 2022). Similarly, Metomura's 2008 study examined anti‐apoptotic proteins and apoptosis in U937 cells treated with Propolis. *BAX* expression remained stable, whereas *Caspase‐3* expression increased and *BCL‐2* expression decreased. These findings showed that Propolis induced apoptosis in U937 cells and that this effect was associated with loss of mitochondrial membrane potential, reduced *BCL‐2* expression, and activation of *Caspase‐3* (Motomura et al. 2008).

Overall, changes in gene expression represent relative shifts in ratios. According to Metomura's study, even in the absence of a change in *BAX* expression after treatment, an altered *BAX*/*BCL‐2* ratio was associated with apoptosis. However, this pattern did not persist at higher concentrations. The current findings of increased *BAX* expression and decreased *BCL‐2* expression are consistent with this interpretation.

Propolis, a natural flavonoid‐rich compound with two important properties, radiosensitivity and radioprotection, has attracted attention as an adjunct to chemotherapy and radiotherapy. This compound acts through different mechanisms in normal and cancer cells. Propolis scavenges free radicals and protects healthy tissues from radiation damage (Vesna Benkovic et al. 2009). It may also act as a radiation attenuator, delaying the onset or reducing the severity of radiation‐induced adverse effects. In cancer cells, its radiosensitizing property enhances anti‐tumor effects when combined with radiation and increases cancer cell death. Activation of *NF‐kB* promotes DNA repair and delays programmed cell death. Propolis can inhibit NF‐κB binding to DNA and disrupt this pathway. By inhibiting *NF‐kB*, NP may increase radiosensitivity by impairing DNA repair (Khoram et al. 2016).

Consistent with these properties, this study evaluated radiosensitizing and radioprotective effects using the alkaline comet assay. To assess radiosensitivity, A‐375 cells were first treated with the IC_50_ concentrations of PE and NP, then exposed to photon (X‐ray) and electron beams. Qualitative and quantitative comparisons of DNA strand breaks before and after irradiation showed that NP, in combination with radiotherapy, significantly increased cancer cell death. This effect was more pronounced for photon irradiation than for electron irradiation (Figure 11).

In Anjaly's 2022 study, the combined effects of CAPE and gamma rays on DU145 and PC3 cells were examined. Alkaline comet assay data showed that CAPE increased γH2AX foci and promoted ionizing radiation‐induced cell death through apoptosis, leading to greater DNA damage and cell mortality. CAPE was proposed as a potential adjunct for the treatment of hormone‐resistant, radiation‐resistant prostate cancer (PCa) (Anjaly and Tiku 2022).

Masoudi et al. examined the MDA‐MB‐231 (estrogen receptor‐negative) and D47T (estrogen receptor‐positive) cell lines. Their alkaline comet assay results showed that CAPE more effectively prevented radiation‐induced DNA damage in D47T cells than in MDA‐MB‐231 cells. These findings suggest that CAPE interferes with DNA damage repair immediately after radiation exposure. Increased radiosensitivity in radiation‐resistant breast cancer cells treated with CAPE may result from persistent DNA damage (Khoram et al. 2016). The results of these studies support the findings of the present work.

In the next step, normal Vero cells were used to investigate radioprotection. Before irradiation, a viability assay confirmed that PE and NP at their IC_50_ concentrations did not exert toxic effects on Vero cells (Figure 3), with no significant difference relative to the control group. Cells were then assessed qualitatively and quantitatively after treatment with PE or NP and exposure to photon or electron beams. Before irradiation, consistent with the viability test, no DNA breaks or significant comet tails were observed, indicating the non‐toxicity of these two compounds in normal Vero cells.

In Vero cells, irradiated samples showed reduced DNA breakage and shorter comet tails than control samples exposed to radiation alone. When comparing the radioprotective effects of PE and NP under photon exposure, NP produced a significantly greater reduction in DNA damage than PE. Under electron exposure, this difference was not significant (Figure 13).

In Benkovic et al. (V Benkovic et al. 2008), the effects of PE and CAPE on white blood cells of CBA mice exposed to whole‐body gamma irradiation were examined. Administration of Propolis and quercetin before irradiation protected white blood cells from radiation‐induced damage and reduced primary DNA breaks, as shown by the alkaline comet assay. These favorable outcomes suggest that Propolis, quercetin, and gamma irradiation in combination may act as effective, non‐toxic radioprotective agents. However, the exact mechanisms of protection and their impact on DNA repair processes remain unclear (V Benkovic et al. 2008).

In a 2009 study by Benkovic, the potential radioprotective effects of the natural substances WSDP, caffeic acid, and naringin on white blood cells exposed to gamma radiation were evaluated. The flavonoid chrysin was assessed using the alkaline comet assay and structural chromosome aberration analysis. None of the tested compounds showed significant genotoxicity, and all of them provided considerable protection against DNA damage (Vesna Benkovic et al. 2009).

## 5 Conclusion

This study demonstrates the anticancer effects of Iranian nanoparticles and their role in enhancing therapeutic efficacy. NP had a lower IC_50_ than PE at 48 h, which indicates a stronger antitumor effect. The nanoparticles measured less than 10 nm and carried a negative surface charge, consistent with long‐term stability.

Analysis of apoptosis‐related genes showed that PE and NP induced apoptosis in cancer cells by altering the expression of pro‐apoptotic genes. The study also examined the radioprotective and radiosensitizing properties of NP and found that NP increased cancer cell death in combination with radiotherapy while sparing normal cells.

Overall, these findings suggest that Propolis, particularly in nanoparticle form, represents a useful natural candidate for cancer treatment.

## Author Contributions

Conceptualization: Nematollah Gheibi, Azam Janati Esfahani; Data curation: Nematollah Gheibi, Azam Janati Esfahani, Nasim Valivand, Hossein Ahmadpour_Yazdi, Hajie Lotfi; Formal analysis: Nasim Valivand, Shima Bidabad, Kimia Taebi, Maryam Karimi; Funding acquisition: Azam Janati Esfahani; Investigation: Nematollah Gheibi, Azam Janati Esfahani, Nasim Valivand; Methodology: Nematollah Gheibi, Azam Janati Esfahani, Hossein Ahmadpour_Yazdi, Nasim Valivand; Project administration: Nematollah Gheibi, Azam Janati Esfahani; Resources: Nematollah Gheibi, Azam Janati Esfahani; Software: Nasim Valivand; Supervision: Nematollah Gheibi, Azam Janati Esfahani; Validation: Nematollah Gheibi, Azam Janati Esfahani; Visualization: Nematollah Gheibi, Azam Janati Esfahani, Nasim Valivand, Hossein Ahmadpour_Yazdi, Hajie Lotfi; Writing – original draft: Nasim Valivand; Writing – review and editing: Nematollah Gheibi, Azam Janati Esfahani.

## Funding

This work was supported by Qazvin University of Medical Sciences, 400000449.

## Ethics Statement

The authors have nothing to report.

## Data Availability

The data that support the findings of this study are available on request from the corresponding author.
